# National Quarantine

**Published:** 1900-05

**Authors:** 


### National Quarantine.

SENATOR VEST has before Congress a bill to enlarge the powers and scope of the Marine Hospital Service in the matter of quarantine, and we learn that there is a fair chance of its passing. It is given out that the Texas authorities are opposing it, and that the Governor has telegraphed the Texas Senators to oppose any interference with State control. This is a matter of great importance to Texas and the South, and for the information of our readers we give a *resumé* of the features of that bill. (Abstracted by *Medical Record.*)

The bill amends a section of the law of 1893 so as to provide that upon the arrival of an infected vessel at any port not provided with proper facilities, the Secretary of the Treasury may send her to the nearest national or other quarantine where proper facilities exist, and, after the treatment of an infected vessel (or the inspection of a vessel not infected) at a national quarantine station, she shall receive a certificate which shall entitle her to entry at any port named in the certificate.

At any port where sufficient quarantine provisions have been made by State or local authorities, the Secretary of the Treasury may direct vessels bound for that port to undergo quarantine at the State or local station.

The bill adds also a very important section to the present law—empowering the supervising surgeon-general of the Marine Hospital Service, with the approval of the Secretary of the Treasury, to designate and mark the boundaries of quarantine grounds and anchorages, and officers of vessels or other persons trespassing on such grounds or anchorages are to be subject to arrest and to a fine of not more than $300, or imprisonment for not more than one year, or both.

There is nothing in the law forbidding the establishment of a State quarantine, notwithstanding a national quarantine be established, and this will only prevent the annoyance of a subsequent inspection, with a charge for the same, by the local quarantine, after the vessel has been thoroughly inspected and given a certificate by the national quarantine officer.

The provision relating to vessels arriving within the limits of any collection district of the United States without a bill of health, is meant to apply particularly to fishing smacks and other small craft which leave Havana and other Cuban ports, and, under the guise of fishing, come within the collection districts of Florida and there land any sick who may be aboard, and smuggle aguardiente, rum, and tobacco, thus holding communication with the shore and being liable to convey infection. These vessels are particularly dangerous, inasmuch as they are manned by laborers recently from Spain and who are, therefore, not immune to yellow fever, and lie in the portions of Havana harbor particularly exposed to yellow fever contagion. The effect of this section will be not only to prevent the introduction of contagious disease, but also to suppress smuggling.

Another section authorizes the Secretary of the Treasury to establish quarantines at points of danger, either on the coast or on the Mexican or Canadian borders. The necessity of having this authority is self-evident. Under the present law, to establish a quarantine requires either that there should be no State quarantine regulations in existence, or the demonstration and proof of inability or negligence to enforce such regulations as the Secretary of the Treasury has made, meanwhile leaving opportunity for disease to enter while the subject is under investigation or dispute.

Aside from the good features of the Marine Hospital bill already noted, it is gratifying to know that no attempt is made to legislate on any matters save those that relate to maritime and border inspection. Altogether it would appear that the latter bill, being the simplest and most practical one, has the best chance for its passage.

---

THE PRESIDENT-ELECT.—Dr. B. E. Hadra, of Waco, late of San Antonio, who was elected President of the Texas State Medical Association at the thirty-second annual meeting at Waco, on 27th April, ult., is a representative of the best element of the medical profession of the State. He is a scholar of wide and varied attainments, an ethical physician, and a writer of international repute. The compliment paid his friends in thus conferring upon him the highest office within the gift of the Association is much appreciated. Dr. Hadra is one of the strong men of the profession. He has exemplified in his life the ideal of the conscientious and ethical physician. In this instance the office unquestionably sought the man. Dr. Hadra has contributed many valuable papers to the literature of surgery and medicine, and is the author of a book on "Lesions of the Pelvic Floor," a work which attracted much attention, especially in Germany, his native land. He has been a citizen of Texas thirty years, and is fifty-seven years of age.

We hope to present our readers a photo-engraving of the President next issue, with a more extended biography.

